# Real-world Independent Testing of e-ASPECTS Software (RITeS): statistical analysis plan

**DOI:** 10.12688/amrcopenres.12904.1

**Published:** 2020-04-28

**Authors:** Grant Mair, Francesca Chappell, Chloe Martin, David Dye, Philip M. Bath, Keith W. Muir, Rüdiger von Kummer, Rustam Al-Shahi Salman, Peter A. G. Sandercock, Malcolm Macleod, Nikola Sprigg, Philip White, Joanna M. Wardlaw

**Affiliations:** 1 Centre for Clinical Brain Sciences, University of Edinburgh, Edinburgh, EH16 4SB, UK; 2 Stroke Trials Unit, University of Nottingham, Nottingham, NG5 1PB, UK; 3 Institute of Neuroscience & Psychology, University of Glasgow, Glasgow, G51 4TF, UK; 4 Department of Neuroradiology, University Hospital Dresden, Dresden, 01309, Germany; 5 Institute of Neuroscience, Newcastle University, Newcastle upon Tyne, NE2 4AX, UK

**Keywords:** stroke, CT, machine learning, automated assessment

## Abstract

**Background:** Artificial intelligence-based software may automatically detect ischaemic stroke lesions and provide an Alberta Stroke Program Early CT score (ASPECTS) on CT, and identify arterial occlusion and provide a collateral score on CTA. Large-scale independent testing will inform clinical use, but is lacking. We aim to test e-ASPECTS and e-CTA (Brainomix, Oxford UK) using CT scans obtained from a range of clinical studies.

**Methods:** Using prospectively collected baseline CT and CTA scans from 10 national/international clinical stroke trials or registries (total >6600 patients), we will select a large clinically representative sample for testing e-ASPECTS and e-CTA compared to previously acquired independent expert human interpretation (reference standard). Our primary aims are to test agreement between software-derived and masked human expert ASPECTS, and the diagnostic accuracy of e-ASPECTS for identifying all causes of stroke symptoms using follow-up imaging and final clinical opinion as diagnostic ground truth. Our secondary aims are to test when and why e-ASPECTS is more or less accurate, or succeeds/fails to produce results, agreement between e-CTA and human expert CTA interpretation, and repeatability of e-ASPECTS/e-CTA results. All testing will be conducted on an intention-to-analyse basis. We will assess agreement between software and expert-human ratings and test the diagnostic accuracy of software.

**Conclusions:** RITeS will provide comprehensive, robust and representative testing of e-ASPECTS and e-CTA against the current gold-standard, expert-human interpretation.

## Introduction

Accurate and rapid identification and quantification of CT imaging features indicative of early ischaemic and haemorrhagic stroke is required to correctly triage patients for urgent treatment. However, early ischaemic brain changes can be subtle on non-enhanced CT (NECT) and identification requires training and experience^1^. The Alberta Stroke Program Early CT Score (ASPECTS) helps quantify early ischaemia on CT and may aid decision making prior to thrombolysis and thrombectomy^2^–^4^. It is particularly important to exclude acute intracranial haemorrhage as the cause of stroke prior to thrombolytic therapy since thrombolysis may worsen haemorrhage. Additionally, a CT angiogram (CTA) may be required immediately after NECT to identify patients with arterial obstruction who are suitable for thrombectomy. Interpretation of CTA also requires training and experience.

A potential solution to help interpret brain CT and CTA after suspected stroke is offered by e-ASPECTS and e-CTA diagnostic software (Brainomix Ltd, Oxford UK). This software, developed using machine learning, includes an automated ASPECTS and detection of acute haemorrhage on NECT, and a CTA assessment to detect large vessel obstruction (LVO) and collateral blood supply. e-ASPECTS is said to be as accurate as expert human rating of ASPECTS^5^,^6^, and the software has been promoted to support and accelerate stroke treatment decisions. However, as is the case with many diagnostic tools developed using machine learning^7^, e-ASPECTS lacks independent demonstration of reliability, safety or clinical utility, especially for newer features of haemorrhage detection and CTA assessment^8^. It is also unclear how e-ASPECTS handles other pathologies which make up around 20% of patients presenting with stroke-like symptoms (i.e. stroke mimics) such as tumours, subdural haematomas or infections.

The Real-world Independent Testing of e-ASPECTS Software (RITeS) study will independently evaluate the accuracy, reliability and clinical benefit of e-ASPECTS and e-CTA software. Here we pre-specify the statistical analysis plan for RITeS.

## Methods

### Study design

We will test the accuracy of e-ASPECTS and e-CTA software for the automated assessment of CT scans performed acutely for suspected stroke among representative patients. We will compare e-ASPECTS/e-CTA with expert human readers who form the current reference standard. We will use CT scans from acute stroke trials that include a range of commonly encountered scan appearances including early ischaemia, haemorrhage, typical pre-stroke features and mimics. All scans have been rated by panels of experts representing many different individuals across these non-commercial trials. We will in addition, perform new human-ratings for a subgroup of the previously collected scans to compare the time needed for human versus e-ASPECTS assessment of CT and to assess the clinical impact of e-ASPECTS software on acute stroke care, i.e. whether it influences diagnostic confidence or alters treatment decisions.

### Patient population

A clinically representative sample (equivalent to the patient population for whom e-ASPECTS or e-CTA may routinely be used) of baseline NECT brain and CTA scans will be selected from the following 10 multicentre national and international randomised-controlled trials (RCTs) and observational studies of stroke: Alteplase versus tenecteplase for thrombolysis after ischaemic stroke (ATTEST)^9^; European multicentre, randomised, phase III clinical trial of therapeutic hypothermia plus best medical treatment versus best medical treatment alone for acute ischaemic stroke (EuroHyp-1)^10^; Third International Stroke Trial (IST-3)^11^; Lothian study of INtraCerebral Haemorrhage Pathology, Imaging and Neurological outcome (LINCHPIN)^12^; Pragmatic Ischaemic Thrombectomy Evaluation (PISTE)^13^; POst-Stroke Hyperglycaemia (POSH)^14^; Penumbra and Recanalisation Acute Computed Tomography in Ischaemic Stroke Evaluation (PRACTISE)^15^; REstart or STop Antithrombotics Randomised Trial (RESTART)^16^; Rapid Intervention with Glyceryl trinitrate in Hypertensive stroke Trial-2 (RIGHT-2)^17^; Safe Implementation of Treatments in Stroke (SITS) open study^18^. These trials collectively include over 6600 individual patients accessible to RITeS; see Table 1 for individual trial numbers and details of available imaging.

All of these trials individually obtained research ethics committee approval. Consent was acquired from or on behalf of all recruited patients.

Baseline CT scans in these trials were scored using very similar methods (often using identical pre-validated schema, i.e. five of the trials used IST-3 methodology for scan assessment^19^, all studies of ischaemic stroke included ASPECTS scoring), and similar patient demographic baseline and outcome data were collected. Scan ratings were obtained by expert readers, nominated by the trials, without knowledge of any clinical baseline or follow-up data, follow-up scans or treatment. Expert rating included assessment for: ASPECTS^2^; acute ischaemia in brain regions other than the MCA territory^1^,^20^; acute intracranial haemorrhage^21^; structural stroke mimics, pre-stroke brain changes (brain atrophy, leukoaraiosis, old stroke lesions)^3^ and image quality. Baseline CTA scans were scored for: arterial obstruction location and extent^22^; collateral extent^23^. 

All of these trials recorded patient demographics (e.g. age, sex), time from stroke onset, included some measure of stroke severity -National Institutes of Health Stroke Scale (NIHSS) for ischaemic stroke, and Glasgow Coma Scale (GCS) for haemorrhagic stroke, and assessed clinical outcome at 90 days or later.

### Inclusion and exclusion criteria

We will derive separate but overlapping samples for e-ASPECTS and e-CTA testing from all baseline scan data available to RITeS. We will produce a STARD-type (Standards for Reporting Diagnostic Accuracy Studies) flow chart^24^ to show reasons for inclusion/exclusion of cases and successful/non-successful scan assessment by human-readers and software for all patients available to RITeS.

***Sample for e-ASPECTS evaluation.*** There are no precise data for the true case-mix of patients initially assessed for presumed stroke, and for whom hospital admission staff may decide to use e-ASPECTS and e-CTA software to assess treatment eligibility. Pooled data from the major thrombolysis and thrombectomy RCTs will be similar to the case-mix considered for these treatments but may include patients with more severe stroke than those seen in routine practice and will not include patients who present late^4^,^25^. Conversely, patients with haemorrhagic stroke are most likely to be severely affected by stroke symptoms^26^, and a proportion may be too sick to be appropriately represented in trials. The Sentinel Stroke National Audit Programme (SSNAP) provides routinely collected UK data for all patients ultimately diagnosed with stroke in England, Wales and Northern Ireland and includes many patients with minor symptoms or delayed/uncertain time of presentation who would not routinely be considered for thrombolysis or thrombectomy^27^. Importantly, all of these data sources only include patients after excluding structural stroke mimics. RIGHT-2 recruited patients at first contact with ambulance staff in the community and reported 26% stroke mimics^17^. A prospective single-centre study of patients with suspected stroke following stroke expert review after arrival at hospital found a similar proportion of mimics^28^. Additionally, in up to 7% of patients with stroke, it is only possible to obtain imaging of poor quality due to patient movement, beam hardening artefacts or variable patient orientation in the scanner, all of which may influence the performance of e-ASPECTS and e-CTA software^3^,^11^. Finally, the sensitivity and specificity of diagnostic tests may vary with prevalence^29^. 

To derive a ‘real-world’ dataset from all patients available to RITeS, and simultaneously maximise sample size, we will therefore:

1) Include all structural stroke mimics for e-ASPECTS testing (since this will be far less than 26%, but see planned sensitivity analyses for our primary outcomes below)
2) For the remainder of included patients (i.e. non-mimics), aim for sex and age mix, stroke severity and time since symptom onset similar to SSNAP, pooled thrombolysis/ thrombectomy RCT data, and haemorrhagic stroke registry data.
3) Not exclude imaging based on quality.

The most recent SSNAP annual dataset (Apr 2018-Mar 2019) identified 87,635 patients presenting acutely with stroke and had 93% data completion. Of these patients, 47.8% were female, median age was 77 years, 87.1% of strokes were ischaemic, 12.4% were haemorrhagic (0.4% unknown), and the median NIHSS was 5. For 69.4% of patients, the time of symptom onset was known and the median time from stroke onset to baseline scan in these patients was 4 hours and 2 minutes^30^. A pooled analysis of nine thrombolysis RCTs by the Stroke Thrombolysis Trialists’ Collaborative (STTC) group included 6,756 patients of whom 45% were female, the mean age was 71 years, the mean NIHSS was 12, and the mean treatment delay was 4 hours^25^. A pooled analysis of seven thrombectomy RCTs by the Highly Effective Reperfusion evaluated in Multiple Endovascular Stroke Trials (HERMES) collaboration included 1764 patients of whom 47% were female, the median age was 67 years, the median NIHSS was 17, and the mean time from onset to randomisation was 3 hours^4^. A comprehensive, community-based audit of haemorrhagic stroke in Scotland (The Lothian Audit of the Treatment of Cerebral Haemorrhage, LATCH) included 137 patients with primary haemorrhagic stroke of whom 55% were female, the median age was 79 years, and the median Glasgow Coma Scale at presentation was 13^31^,^32^.

To ascertain the representativeness of the non-mimic cases within the RITeS dataset, we will report demographic and clinical data as per SSNAP, STTC, HERMES and LATCH data (see Table 2). We will report absolute differences, 95% confidence intervals and p-values for comparisons. We will also present continuous data components using overlapping histograms.

To ascertain the representativeness of relevant radiological features in RITeS, we will also report the features listed in Table 3.

***Sample for e-CTA evaluation.*** The main indication for acute stroke CTA currently is to determine if the patient is suitable for thrombectomy; such patients are more likely to have a more severe ischaemic stroke and to be younger than the median age of all stroke. CTA is used at some sites prior to thrombolysis but this is not universal and there is no standard or widely agreed practice. There is also less CTA data available in RITeS for testing. We will therefore include all patients for e-CTA testing from all available RITeS trials where CTA was performed routinely at baseline, i.e. we will not select a representative subsample.

### Sample size considerations

Tests of diagnostic accuracy are difficult to power and benefit from maximising the sample size^33^. In addition, sample size calculations may be inappropriate in this setting^34^. We therefore aim for the largest representative sample possible within available resources. However, to allow direct comparison with previous work, we estimated that to confirm that e-ASPECTS software is non-inferior for an “MCC (Matthews Correlation Coefficient) better than random” (to expert human calculation of ASPECTS using an MR diffusion-weighted imaging reference standard: e-ASPECTS correlation 0.44 versus 0.38 for experts), based on 34 patients^35^, requires a sample size of 494 scans – at 5% non-inferiority limit with 80% power and at 5% significance level. Therefore, we have set the minimum sample size for our primary outcome at 500 unique patient CT brain scans. Up to 31% of scans may fail to give an e-ASPECTS result for technical reasons^36^, therefore we will inflate the minimum sample to 725 scans (i.e. 1/0.69 × 500) to ensure the successfully processed sub-sample includes the requisite minimum 500 scans. However, this minimum will have limited power for subgroup analyses (e.g. on pre-stroke brain changes, by age or stroke severity strata) and the study cited for our power calculation used individual ASPECTS regions rather than individual patients to power their analysis, which due to lack of independence between individual ASPECTS regions, may be flawed^35^. Therefore we will process every available scan to derive the largest possible sample for testing.

### Image processing

All CT scans will be processed by the RITeS team as follows:

1) In DICOM (Digital Imaging and Communications in Medicine) format
2) On the cloud-based e-ASPECTS platform available at https://brainomix.com
3) Anonymised prior to web upload to remove patient identifiers, but retaining original trial identifiers, using modiCAS DICOM anonymizer (Erlangen, Germany).
4) A person, trained in CT scan handling, will manually select the relevant scan for each patient using four criteria:
  I. First CT scan acquired after the patient reached hospital, if more than one available.
  II. Ideally native images (i.e. non-reformatted) acquired axially, but reformatted scans will be accepted if native imaging not available.
  III. The thinnest CT slices if more than one image set available.
  IV. Scans acquired using a soft-tissue kernel.
  At this stage, all scans meeting these criteria will be processed agnostic of patient or imaging characteristics. Scan selection for entry into the representative RITeS dataset will occur only once processing is complete, blind to imaging appearances and software results (see below).
5) Scans will be batched into zip files of 10+ scans and uploaded to the Brainomix cloud-based platform.
6) Affected side information will not be included at this processing stage but will be entered separately later for a proportion of cases.
7) Any scan upload or processing failures will be recorded in a spreadsheet.
8) Results will be exported from the Brainomix platform, using the standard export function and stored securely offline in .csv format.

### Primary outcomes

1. Difference in ASPECT scores provided by e-ASPECTS versus expert human readers.
2. Diagnostic accuracy of e-ASPECTS versus expert human readers for identifying the cause of stroke symptoms (i.e. ischaemia, haemorrhage, other).

### Secondary outcomes

1. Proportion of scans successfully versus unsuccessfully processed by e-ASPECTS (i.e. where ASPECTS results are provided or not provided), factors associated with e-ASPECTS processing success and result accuracy.
2. Proportion of ICA-MCA occlusion versus normal ICA-MCA axis correctly identified by e-CTA, accuracy of e-CTA for detecting any intracranial obstruction, difference in CTA collateral scores provided by e-CTA versus expert human readers.
3. Repeatability of e-ASPECTS and e-CTA results on a subset of scans presented twice.

### Tertiary outcomes

1. Difference in haemorrhage size and location results provided by e-ASPECTS versus expert human readers.
2. Potential clinical impact of e-ASPECTS and e-CTA on routine acute stroke care, whether:
  a. knowledge of ASPECTS conveyed using e-ASPECTS visual overlay on imaging influences diagnostic confidence or alters treatment decisions,
  b. use of e-ASPECTS/e-CTA alter the time required for scan interpretation, and
  c. e-ASPECTS or e-CTA results are associated with outcome after ischaemic stroke.

### Dissemination of results

We will include all primary and secondary outcomes in the primary RITeS publication.

Tertiary outcomes require additional expert-human data to be collected and will therefore be reported separately, subsequent to the primary RITeS publication.

## Statistical analysis

### Analysis principles and general considerations

- Primary and where appropriate, secondary outcome testing will be conducted on an ‘intention-to-analyse’ basis irrespective of whether scan processing was successful or not
  ◦ Processing will be considered successful when an ASPECTS (0-10) or CTA result (occlusion/no-occlusion or scalar collateral score) is provided, or when arterial hyperattenuation or haemorrhage is detected.
  ◦ We will record all software failures whether these occur at the scan upload, scan processing, or results output stage.
- We will use mean (standard deviation, SD) or median (inter-quartile range, IQR) to represent the distribution of parametric and non-parametric data, respectively. We will use n (%) for categorical data.
- Where possible, we will use diagnostic accuracy statistics as the principal method in RITeS for comparing software and expert human results. With expert human results as the reference standard, we will calculate true/false positive/negative cases (TP, FP, TN, FN, respectively) and derive sensitivity (TP/TP+FN), specificity (TN/TN+FP), positive predictive (TP/TP+FP), negative predictive (TN/TN+FN), and accuracy (TP+TN/TP+TN+FP+FN) percentages as standard. We will calculate 95% confidence intervals for each using the Wilson score method^37^.
  ◦ For all diagnostic accuracy testing we will include random-effects meta-analysis modelling of individual patient data (i.e. a one-step meta-analysis) to provide overall estimates of sensitivity and specificity, to assess variation within and between the 10 RITeS studies, and to account for clustering of individual study results^38^,^39^.
  ◦ For each of the 10 RITeS studies, we will use the PROBAST method for assessing risk of bias in diagnostic modelling^40^.
- For all comparisons of software and expert human results, we will review side of affected brain data to ensure ASPECTS and CTA results are correctly matched for each cerebral hemisphere, i.e. matched results require same score and side. We will separately test whether knowledge of affected side impacts the accuracy of e-ASPECTS results, see secondary outcomes below.
- Bland-Altman plots^41^ will be used to visually compare software and expert human results, comparing mean score differences to assess the magnitude, direction and distribution of error and will use ± 1.96 SD to determine the range of agreement^42^. For software and expert human results to be considered equivalent, we will set maximum clinically acceptable limits of agreement for individual scores, specified in the relevant sections below.
- Krippendorff’s Alpha^43^ (K-alpha) will be used to assess reader (human-human and human-software, as necessary) agreement. K-alpha is generally more robust than kappa in this context since it can handle categorical and ordinal data, works where there is missing data, adjusts for small sample sizes, and includes multiple observers simultaneously^43^,^44^. K-alpha results are interpreted similarly to kappa with scores ranging from -1 (perfect disagreement) to +1 (perfect agreement). We will therefore also use the Landis and Koch method for interpreting K-alpha results (as commonly used for kappa): 0.00–0.20=slight agreement, 0.21–0.40=fair agreement, 0.41–0.60=moderate agreement, 0.61–0.80=substantial agreement and 0.81–1.00=almost perfect agreement^45^.
- Other specific statistical tests are listed in the following sections.
- For multivariable testing, we will check for multicollinearity between included variables by identifying variance inflation factors (VIF) >5. Where multicollinearity is detected, we will run separate (but otherwise equivalent) multivariable regression models for each of the internally correlated variables.
- We will not impute but will report missing data (proportions of data missing for each variable and observations not included in analyses).
- We will use SPSS, IBM Corporation (Armonk, USA) and/or R (https://www.r-project.org/) statistical software for all analyses. We will preferentially report 95% confidence intervals, but where appropriate, we will report p-values.

### Primary outcomes

For these evaluations of e-ASPECTS, we will use a representative RITeS sample of NECT.

In two analyses, we will compare e-ASPECTS results to those provided by expert human readers for the assessment of non-enhanced brain CT acquired at baseline among patients presenting acutely with symptoms of stroke.

***Representative sampling for e-ASPECTS testing of primary outcomes.*** Where clinical or demographic features are different to the comparator SSNAP/STTC/HERMES datasets (i.e. RITeS values should be within the range provided by the other datasets), we will use stratified random sampling to remove select cases (which are over-representing these features) to reduce these differences while maintaining the maximum sample size from the data available to RITeS. For example, if RITeS has 42% female patients and a median NIHSS of 19, we would identify all male patients with a high NIHSS and randomly remove subsets of these until the sex ratio and NIHSS are within the desired ranges. We will not select cases on the basis of the radiological features in Table 3.

To assess the impact of factors beyond our control which might affect the representativeness of our sample, we will perform sensitivity analyses of our primary outcomes for the following randomly selected subgroups:

1. With balanced representation from all RITeS trials, i.e. we will exclude surplus cases for trials which are relatively over-represented (more than double the median trial contribution).
2. Where non-stroke mimics represent 26% of the total sample.

***1. Difference in ASPECT scores of e-ASPECTS and expert human readers.*** We will compare e-ASPECTS and expert human results using overlapping histograms and Mann-Whitney U testing to assess the distribution of results, and Wilcoxon signed rank testing and Bland-Altman plots to assess pairwise agreement for each case. For results to be considered equivalent on Bland-Altman testing, we expect the range of agreement to be within ± 2 ASPECTS points. This is an arbitrarily derived but clinically meaningful difference that might lead to alterations in treatment for individual patients based on the thresholds presented below. We will also use K-alpha statistics to assess software-human reader agreement for these scalar data.

We will classify e-ASPECTS and expert-human scores into three groups to assess diagnostic accuracy at the following clinically relevant cut-points, and present a summary (receiver-operating characteristic) ROC curve:

1) ASPECTS 10 (normal) versus ASPECTS 0-9 (abnormal)
2) ASPECTS 0-7 versus 8-10^2^.
3) ASPECTS 0-5 versus ASPECTS 6-10^4^.

To compare with previous work, we will use Matthews correlation coefficient (MCC), and test non-inferiority between e-ASPECTS and expert-human scores^5^,^35^. We will calculate MCC as (TPxTN)-(FPxFN)/√(TP+FP)(TP+FN)(TN+FP)(TN+FN)^46^. We will set our non-inferiority margin at 5%. Thus using the two one-sided test (TOST) procedure, we will establish non-inferiority, using the following formula: at the α=5% significance level, if the lower limit of a (1-2α) × 100% (i.e. 90%) confidence interval for the difference (e-ASPECTS minus expert-human results) is above -5%. The confidence interval is set at (1-2α) rather than the usual (1-α) because the method is equivalent to performing two one-sided tests^47^,^48^.

***2. Accuracy of e-ASPECTS versus expert human readers for identifying cause of stroke symptoms.*** We will compare the diagnostic accuracy of e-ASPECTS software and expert human readers (at baseline, blind to all other clinical data including further imaging) for three groups against the reference standard, human-expert opinion using all available follow-up data including further imaging:

1) Identifying features of ischaemic stroke (including ischaemic lesions in any brain location and/or intracranial hyperattenuating arteries)
2) Identifying haemorrhage, and
3) Identifying any structural causes of stroke symptoms on baseline imaging (including mimics).

In addition, to aid clinical understanding and real-world applicability, we will summarise all primary outcome results using normalised frequencies. Thus we will present results as proportions of 1000 individual patients, i.e. a test sensitivity of 90% would be presented as: the test will detect disease in 900 of 1000 patients with the disease, but 100 patients with the disease will be missed by the test^49^.

### Secondary outcomes

***1. e-ASPECTS processing success and factors influencing accuracy.*** To present the most complete picture available, we will use the entire RITeS sample for these analyses (i.e. not just the representative sample) but we will also include a sensitivity analysis of the representative sample.

We will report the proportion of scans successfully and unsuccessfully processed by e-ASPECTS and collate reasons cited by e-ASPECTS for any processing failures. We will use summary statistics to describe and compare the subgroups of scans that were successfully versus unsuccessfully processed. This will include patient and radiological factors (see Table 4).

To determine the influence of patient and radiological factors on the accuracy of ASPECTS results produced by e-ASPECTS software, we will perform multivariable ordinal logistic regression using the variables pre-specified in Table 5. The dependent variable will be the absolute difference in ASPECTS between e-ASPECTS and expert human readers (i.e. scalar, 0-10).

For any variables found to be significantly associated with poorer e-ASPECTS results on regression testing (i.e. independently associated with greater difference between e-ASPECTS and expert human reader scores), we will also calculate and compare diagnostic accuracy figures (as above) for each of the subgroup arms (for these analyses only, continuous data will be dichotomised as per Table 5).

Finally, to account for (the as yet unknown) covariates that alter e-ASPECTS diagnostic accuracy figures, we will derive covariate-adjusted ROC curves (AROC)^50^.

***2. Identification of CTA obstruction and collateral scoring by e-CTA versus expert human readers.*** There are two components to the e-CTA output:

1. Where ICA-MCA occlusion is identified, the location is categorised either proximal (ICA/proximal MCA) or distal (distal MCA). Other arteries are not assessed.
2. MCA collateral scoring (modified Tan *et. al*. 2009)^51^ is given for all cases on the scale 0 = no collaterals (<10% of affected MCA territory compared to contralateral side), 1 = poor (10–50%), 2 = good (50–90%), 3 = excellent (>90%) collaterals (i.e. includes normal scans).

The expert human rated CTAs available to RITeS include similar scoring methods for comparison with e-CTA outputs, but also additional measures such as degree of arterial patency (i.e. from fully patent through increasingly obstructed to occluded)^22^.

We will test three components:

1. The proportion of scans where e-CTA and expert humans agree or disagree in the assessment of proximal versus distal ICA-MCA axis occlusion, see Table 6. We will use K-alpha statistics to compare agreement for ICA-MCA axis occlusion detected by e-CTA and expert human readers.
2. Diagnostic accuracy of e-CTA for detecting abnormal versus normal intracranial CTA using the methods described above. Abnormal will include both arterial obstruction (partially blocked) and arterial occlusion (completely blocked) for 11 named intracranial arterial segments, i.e. not just ICA or MCA; we will also assess the anterior and posterior cerebral arteries (ACA and PCA, respectively), the vertebral and basilar arteries (five left, five right, one central).
3. Agreement on collateral score. Most of the expert collateral scoring available in RiTeS used the Miteff method (three-point scalar = good, moderate, poor)^23^, rather than the modified Tan method (4-point scalar = excellent, good, poor, none) but as ordinal scores, these are comparable. There are three ways to compare the scores (see Table 7). We will test all variations for agreement. We will use K-alpha statistics to check the extent of agreement between e-CTA and human-rated results for the scalar collateral scoring.

***3. Repeatability of e-ASPECTS and e-CTA results.*** We will select a small subgroup of RITeS scans for repeat e-ASPECTS and e-CTA testing. These subgroup sizes were arbitrarily chosen within available time and scan processing resource limitations.

We will include separate assessments of ASPECTS (n=100), haemorrhage detection (n=20) and CTA scoring (n=20). We will select scans for repeat testing, blind to all previous results (from e-ASPECTS, e-CTA or expert human readers) except knowledge of previous successful processing by e-ASPECTS or e-CTA. To ensure this subsample remains largely representative of the available stroke trial mix, we will use cluster random sampling, as follows. Each stroke trial represents a cluster. Random samples will be drawn from each cluster in numbers to match individual stroke trial proportions in the entire representative sample until the total numbers required are reached for each of the separate assessment groups, total n=140.

To limit the possibility of previously calculated results being presented again (rather than freshly derived from the ‘new’ scan), selected scans will have all unique identifiers replaced prior to repeat e-ASPECTS/e-CTA processing. This includes original trial IDs and any other DICOM information that uniquely identifies individual scans (e.g. accession number, series/scan unique identifiers).

We will compare original and repeat results for agreement, as per Table 8. We will use Mann-Whitney U tests to compare scalar and non-parametric continuous group data, and K-alpha to compare paired scan results.

### Tertiary outcomes

***1. Haemorrhage quantification by e-ASPECTS versus expert human readers.*** We will use all RITeS NECT containing acute haemorrhage for this analysis. This includes scans acquired from haemorrhagic stroke trials (i.e. brain haemorrhages with or without intraventricular extension) in addition to scans classed as mimics in ischaemic stroke trials (e.g. subdural or subarachnoid haemorrhages).

Haemorrhage will be quantified by location and extent as follows.

*Haemorrhage location*

Haemorrhage location will be defined by side and gross anatomical regions of the brain affected including cerebral lobes (i.e. frontal, parietal, temporal, occipital), basal ganglia, brainstem, cerebellum, or extra-axial compartment (i.e. intra-ventricular, subarachnoid, subdural, extra-dural). We will convert e-ASPECTS haemorrhage detection overlay to these same 22 (11 per side) categories following visual review, blinded to human-reader results.

We will look at differences in the regions (and their number) identified by expert humans and e-ASPECTS and if the data are amenable, consider using methods that account for multiple concurrent haemorrhage sites per patient^52^.

*Haemorrhage extent*

Haemorrhage extent will require a comparison of haemorrhage volume calculated at the voxel level (e-ASPECTS) and haemorrhage dimensions used to estimate volume (i.e. the ABC/2 score^21^,^53^, human readers). We will use Bland-Altman testing (expected range of agreement to be within ± 10 mm^3^) and K-alpha statistics to test agreement between e-ASPECTS and human-rated results.

***2a. Impact of e-ASPECTS on diagnostic confidence and treatment decisions.*** We will invite stroke and hospital admission physicians with a range of experience to complete an online questionnaire including RITeS NECT and relevant clinical scenarios, similar to previous work conducted by RITeS members^1^,^54^. We will compare responses for readers before and after ASPECTS is calculated (i.e. with and without e-ASPECTS overlay) to assess whether ASPECTS alters confidence in stroke diagnosis and determine its influence on management decisions.

RITeS cases with the following range of relevant radiological findings will be selected: with obvious, subtle and no ischaemic brain lesions (and a range of ASPECTS results); with and without hyperattenuating arteries; with subtle brain and extra-axial haemorrhage; with and without pre-stroke brain changes.

The questionnaire will contain 24 cases including some repetition of the same NECT with and without e-ASPECTS overlay. Specifically, we will include 10 cases shown with and also without e-ASPECTS overlay. To limit reader recognition of repeat cases, these images will be modified (e.g. left-right reversal, removal of any visible extracranial image components) and presented in a non-sequential order. We will also include two unique cases with and two unique cases without e-ASPECTS overlay; each of these 4 cases will be displayed only once. Thus 12 cases will be presented with and 12 without e-ASPECTS overlay. A unique clinical history will be provided for all 24 cases but we will limit clinical variability between repeat cases with and without e-ASPECTS overlay. Each case will include a panel of JPEG images representing the whole brain. Repeat images with and without e-ASPECTS overlay will include identical slices.

We will include up to three questions for each case:

1. Is the e-ASPECTS overlay helpful?
  • Yes
  • No
2. Given the clinical history and the images presented, would you
  • Treat the patient immediately with IV alteplase (+/- refer for thrombectomy where available)
  • Refer the patient immediately for thrombectomy without IV alteplase
  • Not treat the patient with IV alteplase or thrombectomy but transfer to the acute stroke ward
  • I’m not sure, I’d first ask for a neuroradiology opinion
  • I’m not sure, I’d like to do more imaging such as angiography, perfusion imaging or MRI (not available here)
3. How confident are you with this decision?
  • Very unsure
  • Unsure
  • Sure
  • Very sure

We will present these results as per Table 9, and visually in bar charts. We will compare results for the following subgroups in univariable analysis (chi-squared statistics): with versus without e-ASPECTS overlay, more versus less experienced readers, obvious versus subtle or no ischaemic lesion, with versus without haemorrhage, with versus without hyperattenuated arteries, with versus without leukoaraiosis, atrophy or old stroke lesions.

***2b. Use of e-ASPECTS/e-CTA and time required for scan interpretation.*** We will select 100 NECT and 50 CTA from all RITeS cases that have been successfully processed by e-ASPECTS or e-CTA software, respectively and where expert human and software results match (to limit the likelihood that one test group is disadvantaged by known or unknown factors that make scan reading more difficult for either group). We will use stratified random sampling to ensure relevant scan appearances are equally represented as follows:

- From all available NECT with valid e-ASPECTS result we will create three strata
  i. No acute ischaemic lesion (i.e. ASPECTS = 10)
  ii. Small acute ischaemic lesion (ASPECTS 6-9)
  iii. Medium-large acute ischaemic lesion (ASPECTS 0-5)
- From all available CTA with valid e-CTA result we will create three strata
  i. No arterial obstruction
  ii. Proximal ICA-MCA obstruction
  iii. Distal MCA obstruction

We will randomly sample similar numbers (i.e. one-third) from each of the three NECT and CTA strata (therefore ~33 cases for each NECT stratum and ~16 for each CTA stratum).

We will examine the performance of e-ASPECTS/e-CTA software versus:

a. Radiology/stroke physician trainees, front-of-house clinicians (non-experts)
b. Experienced stroke physicians or neuroradiologists (experts)

We aim to include a minimum of five expert and five non-expert readers. Each reader will be shown a unique random 10% selection (10 NECT and 5 CTA) of the cases on a PACS (picture archiving and communication system) workstation suitable for clinical review of DICOM imaging. If more than 10 readers are recruited to the study, we will allow repeat reading of cases. Readers will be asked to fully evaluate CT and CTA as required for routine stroke care using a standard proforma. Full evaluation will include assessment for all potential causes of stroke symptoms in any intracranial location (NECT – ischaemic brain lesion including ASPECT scoring, hyperattenuating artery sign, haemorrhage, mass lesion) and identification of arterial obstruction that might cause ischaemic stroke (CTA – including collateral scoring when relevant). All scan ratings will be performed blind to clinical characteristics, prior human reading and prior e-ASPECTS/e-CTA software results.

An observer will record the time taken (in seconds) for full NECT and CTA evaluation, as well as the time needed for ASPECT and CTA obstruction scoring alone. Software times will be measured from initiation of the software prior to scan loading to receipt of a valid output. Valid outputs include either an ASPECT score, identification of a hyperattenuated artery, or determination of arterial patency (i.e. to ensure a fair comparison, software errors will be excluded from this analysis).

We will compare the time needed for human (all, expert only, non-expert only) and software derived ASPECTS and CTA obstruction/collateral scoring separately in univariable analyses, i.e. Mann-Whitney U testing.

### 2c. Association between e-ASPECTS or e-CTA results and clinical outcome after stroke

We will use the entire RITeS samples of NECT and CTA for these analyses but also perform sensitivity analyses using the representative NECT sample.

In separate multivariable analyses, we will test whether the three main software outputs of e-ASPECTS (ASPECTS result) and e-CTA (ICA-MCA axis occlusion, MCA territory collateral score) are independently associated with stroke outcome. We will include the following variables in each model since these are already known to be associated with outcome after stroke: age, NIHSS, time from stroke onset, treatment with alteplase and/or thrombectomy (vs no treatment). The dependent variable in each model will be functional outcome after stroke. Assessment of functional outcome after stroke varies among the RITeS trials and includes either the modified Rankin Score (mRS) at 90 days, or the Oxford Handicap Scale (OHS) at 6 months from stroke onset. Both mRS and OHS are 7-point scalar ranging from normal (0) through increasing disability (1–5), to death (6). We will use multivariable ordinal logistic regression to calculate common odds ratios for good outcome, presented as per Table 10.

### Future data availability

In general, clinical imaging data in DICOM format are difficult to fully anonymise and are not routinely available for open sharing. Other clinical trial data may be available.

When RITeS data become available, these will be included here:

https://datashare.is.ed.ac.uk/handle/10283/3105. Some of the individual trial data are separately available as follows: IST-3 - https://datashare.is.ed.ac.uk/handle/10283/1931; RESTART - https://datashare.is.ed.ac.uk/handle/10283/3265.

## Conclusions

RITeS will provide robust but fair independent testing of e-ASPECTS and e-CTA software from Brainomix measured against the current gold standard for CT imaging assessment, expert-human interpretation.

This statistical analysis plan pre-specifies all methods prior to un-blinding and analysis of RITeS data.

## Data availability

### Underlying data

No underlying data are associated with this article.

### Reporting guidelines

Edinburgh Datashare: Statistical Analysis Plan checklist for ‘ Real-world Independent Testing of e-ASPECTS Software (RITeS): statistical analysis plan’. https://doi.org/10.7488/ds/2803^55^.

The completed reporting guidelines checklist is available under the terms of the Creative Commons Attribution 4.0 International license (CC-BY 4.0).

## Statement of independence

The authors and wider RITeS study research team declare that Brainomix Ltd, their staff and other affiliated individuals have not been involved in the creation of this research plan or the setting of the RITeS aims and objectives. Image processing, analysis, interpretation and dissemination of results will be conducted independent of Brainomix Ltd and its affiliates.

## Figures and Tables

**Table 1.** Clinical stroke trials with baseline CT scans available for inclusion in RITeS.

Stroke Trial	Number patients	Baseline CT Assessment	Stroke Type	Trial Type	Other Scans	Follow-up Data
ATTEST	104	ASPECTS	Isch	RCT – IV alteplase or tenecteplase	CT at 24–48 hrs	2 & 90 days
EuroHyp	98	As per IST-3, excluding CTA.	Isch	RCT – systemic cooling to 34-5°C for 24 hours or control	CT/MRI at day 5	0–7 & 90 days
IST-3	3035	Ischaemia extent & location, ASPECTS, swelling, atrophy, leukoaraiosis, old stroke lesions, arterial patency & collateral scores on CTA, scan quality 3.	Isch	RCT – IV alteplase or control	Baseline CTA (in ~270) CT/MRI at 24–48 hrs	7 days & 6- months
LINCHPIN	947 *	Location & size of intracerebral haemorrhage	Haem	Observational cohort study	MRI at 3 months	Post-mortem
PISTE	65	As per IST-3.	Isch	RCT – IV alteplase and thrombectomy or IV alteplase alone	Baseline CTA CT/MRI at 22–36 hrs	2 & 90 days
POSH	113	Ischaemia volume on CT, ASPECTS, arterial patency on CTA	Isch	Observational study	CT at 24–48 hrs	
PRACTISE	271	As per IST-3.	Isch	RCT – Multimodal CT imaging (CT + CTA + CTP) or CT alone	Baseline CTA (in 50%) CT/MRI at 24 hrs	1, 7 & 90 days
RESTART	537	Location & size of haemorrhage, atrophy, leukoaraiosis, old stroke lesions, scan quality	Haem	RCT – restart or avoid antiplatelets after haem stroke	MRI pre- randomization (in 550)	1–3 years
RIGHT-2	1149	As per IST-3.	Isch	RCT – ultra-early transdermal GTN or control	CT at 24–48 hrs	4 & 90 days, 1 year
SITS Open	293	ASPECTS, swelling, arterial patency on CTA	Isch	Controlled study -thrombectomy vs control	Baseline CTA (in ~5%) CT/MRI at 22–36 hrs	1, 7 & 90 days

* Ongoing at time of data collection.

**Table 2.** Planned assessment of demographic and clinical data for non-stroke mimic cases within the RITeS dataset, and comparison with other datasets.

Clinical Feature		RITeS dataset	SSNAP dataset	STTC dataset	HERMES dataset	LATCH dataset
Total patient number			87,635	6,756	1,764	137
Female sex			48%	45%	47%	55%
Age, years			77 (66-85)	71 (13)	67 (57-76)	79 (67-83)
Aetiology	Ischaemia		87%	100%	100%	-
	Haemorrhage		12%	-	-	100%
NIH Stroke Scale			5 (2-11)	12 (7)	17 (13-21)	-
Glasgow Coma Scale			-	-	-	13 (9-15)
Time from stroke onset, hours			4 (2-11)	4 (1.2)	3 (2-4)	-

Note: Data are percentage, median (inter-quartile range), or mean (standard deviation) as appropriate.

**Table 3.** Planned assessment of radiological characteristics of the entire RITeS dataset.

Radiological Finding		N (%) or Median (IQR)
Ischaemic brain changes	None	
	Subtle (e.g. loss of grey-white margins)	
	Obvious (e.g. hypoattenuation relative to normal white matter +/- swelling)	
Ischaemic location	MCA territory	
	Other cerebral	
	Brainstem or cerebellum	
	Cortical	
	Subcortical	
Hyperattenuating arteries	List location	
Haemorrhage location	Deep	
	Lobar	
	Intraventricular	
	Extra-axial	
Structural stroke mimic	List type (e.g. tumour, inflammation, collection)	
Pre-stroke brain changes	Atrophy	
	Leukoaraiosis	
	Old Stroke lesions	
CT slice thickness	Thin (≤ 1 mm)	
	Medium (> 1 mm ≤ 5 mm)	
	Thick (> 5 mm)	

**Table 4.** Planned univariable comparison of scans successfully versus unsuccessfully processed by e-ASPECTS.

Variable	Successfully Processed	Not Successfully Processed	Absolute differences, 95% CI and p-value
Patient age (median, IQR)			
Patient sex (n, %)			
Minutes from stroke onset to scan (median, IQR)			
NIHSS (median, IQR)			
Stroke aetiology (n, %): • Ischaemia MCA territory • Ischaemia elsewhere • Haemorrhage • Mimic			
Presence of pre-stroke brain changes (n, %): • Atrophy • Leukoaraiosis • Old stroke lesions			
CT slice thickness (n, %): • ≤ 1 mm • > 1 mm ≤ 5 mm • > 5 mm			
Image quality (n, %): • Good quality • Movement artefacts • Streak artefacts • Patient malposition			

Note: IQR = Inter-quartile range. MCA = middle cerebral artery. NIHSS = National Institutes of Health Stroke Scale.

**Table 5.** Variables for multivariable analysis and subgroups for diagnostic accuracy testing.

Clinical Characteristics		Imaging Findings		Imaging Technique	
Age	≤60 years	ASPECTS	<6	CT slice thickness	≤1 mm
	>60 years		≥6		> 1 mm
NIHSS	0–6	Infarct Location	MCA territory	Poor patient positioning	Yes No
	7–12		Elsewhere		
	>12	Atrophy, Leukoaraiosis, Old stroke lesion	Yes No		
Time from stroke onset to scan	<3 hours				
	3+ hours			Imaging artefact (movement or streak)	
Knowledge of affected side	Yes No				

**Table 6.** Comparison of ICA-MCA axis occlusion identified by e-CTA and expert-human groups.

Human Readers e-CTA	Proximal Occlusion	Distal Occlusion	TOTAL
Proximal Occlusion			
Distal Occlusion			
**TOTAL**			

**Note:** All results n (%). Proximal = ICA or proximal MCA (M1). Distal = distal MCA (M2+).

**Table 7.** Options for comparing modified Tan (e-CTA) and Miteff methods (human rated) of MCA collateral scoring.

Modified Tan used by e-CTA to grade collaterals	Miteff comparison 1	Miteff comparison 2	Miteff comparison 3
3 = Excellent (>90%)	Good	Good	Good
2 = Good (50–90%)		Moderate	Moderate
1 = Poor (10–50%)	Moderate		Poor
0 = None (0–10%)	Poor	Poor	

**Table 8.** Planned results for e-ASPECTS and e-CTA repeatability testing.

Tested Component	Result
e-ASPECTS, ischaemia	N, % with matched ASPECTS results
	N, % for each of 1–10 point ASPECTS difference
	Median, IQR for difference in scores
e-ASPECTS, haemorrhage	N, % with matched location results
	N, % for each of 22 named regions
	Median, IQR for comparison of haemorrhage volumes
e-CTA, obstruction	N, % with matched ICA-MCA proximal vs distal results
e-CTA, collateral scoring	N, % with matched collateral scores
	Median, IQR for comparison of collateral scores

Note: ICA = internal carotid artery. MCA = middle cerebral artery.

**Table 9.** Planned results for effect of e-ASPECTS on diagnostic confidence and treatment decisions questionnaire.

Question	Total, n (%)	Subgroups: e-ASPECTS overlay, reader experience, visible ischaemic lesion, haemorrhage, hyperattenuating arteries, leukoaraiosis, atrophy, old stroke lesions		Absolute differences, 95% CI and p-value
		With n (%)	Without n (%)	
Found e-ASPECTS overlay helpful				
Decision				
Treat patient with thrombolysis/ thrombectomy				
No acute treatment				
Request further radiological input				
Confidence in decision				
Very unsure				
Unsure				
Sure				
Very sure				

Note: Table truncated for presentation of subgroups.

**Table 10.** Planned ordinal regression analysis results with 3–6-month post-stroke outcome as dependent variable.

Variable	Raw Data N (%) or Median (IQR)	Odds Ratio, OR	95% Confidence Interval	p-value
Age, years				
NIHSS				
Time from stroke onset, minutes				
Treated with alteplase +/- thrombectomy				
Software-derived result *				

Note: NIHSS = National Institutes of Health Stroke Scale, IQR = Inter-quartile range. * Either ASPECTS result, presence/absence of ICA-MCA axis occlusion, MCA collateral score.
