# Supplementary material for: Real-world Independent Testing of e-ASPECTS Software (RITeS): statistical analysis plan
Source: AMRC Open Res. Author manuscript; Available in PMC 2022 Jul 6. (PMC7612993; doi:10.12688/amrcopenres.12904.1)
Supplement: Peer review history

# Peer review: Reviewer response for version 1

**DOI:** 10.21956/amrcopenres.13974.r26588

**Published:** 2021-01-27

**Authors:** Hernán Bayona, Nicolás Useche

**Affiliations:** 1 Primary Stroke Center, Neurology Department, University Hospital Fundación Santa Fe de Bogotá, Bogotá DC, Colombia; 2 Primary Stroke Center, Diagnostic Imaging Department, University Hospital Fundación Santa Fe de Bogotá, Bogotá DC, Colombia

Imaging biomarkers have been guiding diagnosis, therapeutic decisions and prognosis in stroke patients for years. After results of NINDS trial, thrombolytic therapy started to include more patients based on increasing time window from 3 hours now up to 9 hours for IV treatment and up to 24 hours for thrombectomy. Moving the imaging paradigm from one third of MCA territory just with visual impression to the Alberta Stroke Program Early CT Score (ASPECTS) to measuring the amount of infarcted and penumbra tissue. After year 2014 and the results of the thrombectomy trials, the collateral status of the patients also gain a place in order to decide when to treat an acute stroke patient. These excellent imaging tools can be automatized and training artificial intelligence models as RITeS protocol based on imaging bases readings from experts from main clinical trials (IST-3, ATTEST, PISTE, RESTART i.e). Using the non-contrast CT (NCCT) results, the ASPECTS score & CTA to indicate arterial occlusion location, severity and collateral status authors from the protocol RITeS, Mair et al, elaborate a complete document to test e-ASPECS and e-CTA (Brainomix) constructed on the imaging bank (expert reading but highly selected subjects), mixed population imaging from studies like HERMES, RIGHT-2 (expert reading but diverse subjects) and finally compared those tests and tools in the real world. Experts (neuroradiologists or stroke neurologists) and non-expert (training personnel) are going to be examined and finally determined accuracy, reliability and concordance of the test supported by the e-technology. It is exciting to find out if clinical decisions are supported and easiest for physicians in the frontline. We consider this protocol a complete document, with robust statistics and planning. Maybe we would explain with more detail if the CTA readings are from source images, (multiple intensity projection) MIP reconstructions or both, and if the observations will include axial, coronal and sagittal images. Otherwise, we cannot see a weakness in the approach to develop the strategy.

Is the study design appropriate for the research question?

Yes

Is the rationale for, and objectives of, the study clearly described?

Yes

Are sufficient details of the methods provided to allow replication by others?

Yes

Are the datasets clearly presented in a useable and accessible format?

Yes

Reviewer Expertise:

Stroke, epidemiology

We confirm that we have read this submission and believe that we have an appropriate level of expertise to confirm that it is of an acceptable scientific standard.

# Peer review: Reviewer response for version 1

**DOI:** 10.21956/amrcopenres.13974.r26580

**Published:** 2020-11-25

**Authors:** Valentina Saia

**Affiliations:** 1 Neurology department/Stroke Unit, Santa Corona Hospital, Pietra Ligure, Italy

Artificial intelligence-based software's are nowadays widely used in acute stroke setting. The authors aim to evaluate the accuracy and clinical benefit of automated e-ASPECTS and e-CTA in clinical practice comparing them with expert human reading. 

 The rationale is well described and the study protocol is clear. The strength of this study includes the number of imaging scans declared, the variety of the sources, and the inclusion of stroke mimics in the case mix. Of particular interest, the tertiary outcome focusing on the possible impact of automated software reading on acute stroke care practice.

 Also, the statistical analysis is described in detail and the ASPECTS cut-off points established for categorizing the population are pragmatic.

Is the study design appropriate for the research question?

Yes

Is the rationale for, and objectives of, the study clearly described?

Yes

Are sufficient details of the methods provided to allow replication by others?

Yes

Are the datasets clearly presented in a useable and accessible format?

Yes

Reviewer Expertise:

Neurosciences, expert in vascular neurology

I confirm that I have read this submission and believe that I have an appropriate level of expertise to confirm that it is of an acceptable scientific standard.
